# Human anogenital distance: an update on fetal smoke-exposure and integration of the perinatal literature on sex differences

**DOI:** 10.1093/humrep/dev323

**Published:** 2016-01-04

**Authors:** Paul A. Fowler, Panagiotis Filis, Siladitya Bhattacharya, Bruno le Bizec, Jean-Philippe Antignac, Marie-Line Morvan, Amanda J. Drake, Ugo Soffientini, Peter J. O'Shaughnessy

**Affiliations:** 1 Institute of Medical Sciences, University of Aberdeen, Foresterhill, Aberdeen AB25 2ZD, UK; 2 Institute of Applied Health Sciences, University of Aberdeen, Foresterhill, Aberdeen AB25 2ZD, UK; 3 USC INRA 1329 Laboratoire d'Etude des Résidus et Contaminants dans les Aliments, LUNAM Université, Oniris,Nantes F-44307, France; 4 Endocrinology Unit, Queen's Medical Research Institute, University/BHF Centre for Cardiovascular Science, University of Edinburgh,47 Little France Crescent, Edinburgh EH16 4TJ, UK; 5 Institute of Biodiversity, Animal Health & Comparative Medicine (IBAHCM), College of Medical, Veterinary & Life Sciences, University of Glasgow, Bearsden Rd, Glasgow G61 1QH, UK

**Keywords:** anogenital distance, endocrine-disrupting chemicals, maternal cigarette smoking human, fetus, normative data, proliferation, apoptosis, second trimester, dysregulation, testosterone

## Abstract

**STUDY QUESTION:**

Do sex and maternal smoking effects on human fetal anogenital distance (AGD) persist in a larger study and how do these data integrate with the wider literature on perinatal human AGD, especially with respect to sex differences?

**SUMMARY ANSWER:**

Second trimester sex differences in AGD are broadly consistent with neonatal and infant measures of AGD and maternal cigarette smoking is associated with a temporary increase in male AGD in the absence of changes in circulating testosterone.

**WHAT IS KNOWN ALREADY:**

AGD is a biomarker of fetal androgen exposure, a reduced AGD in males being associated with cryptorchidism, hypospadias and reduced penile length. Normative fetal AGD data remain partial and windows of sensitivity of human fetal AGD to disruption are not known.

**STUDY DESIGN, SIZE, DURATION:**

The effects of fetal sex and maternal cigarette smoking on the second trimester (11–21 weeks of gestation) human fetal AGD were studied, along with measurement of testosterone and testicular transcripts associated with apoptosis and proliferation.

**PARTICIPANTS/MATERIALS, SETTING METHODS:**

AGD, measured from the centre of the anus to the posterior/caudal root of penis/clitoris (AGD_app_) was determined in 56 female and 70 male morphologically normal fetuses. These data were integrated with current literature on perinatal AGD in humans.

**MAIN RESULTS AND THE ROLE OF CHANCE:**

At 11–13 weeks of gestation male fetal AGD_app_ was 61% (*P*< 0.001) longer than in females, increasing to 70% at 17–21 weeks. This sexual dimorphism was independent of growth characteristics (fetal weight, length, gonad weight). We confirmed that at 14–16 weeks of gestation male fetal AGD_app_ was increased 28% (*P* < 0.05) by *in utero* cigarette smoke exposure. Testosterone levels were not affected by smoking. To develop normative data, our findings have been integrated with available data from *in vivo* ultrasound scans and neonatal studies. Inter-study variations in male/female AGD differences lead to the conclusion that normalization and standardization approaches should be developed to enable confidence in comparing data from different perinatal AGD studies.

**LIMITATIONS, REASONS FOR CAUTION:**

Sex differences, and a smoking-dependent increase in male fetal AGD at 14–16 weeks, identified in a preliminary study, were confirmed with a larger number of fetuses. However, human fetal AGD should, be re-assessed once much larger numbers of fetuses have been studied and this should be integrated with more detailed analysis of maternal lifestyle. Direct study of human fetal genital tissues is required for further mechanistic insights.

**WIDER IMPLICATIONS OF THE FINDINGS:**

Fetal exposure to cigarette smoke chemicals is known to lead to reduced fertility in men and women. Integration of our data into the perinatal human AGD literature shows that more work needs to be done to enable reliable inter-study comparisons.

**STUDY FUNDING/COMPETING INTEREST(S):**

The study was supported by grants from the Chief Scientist Office (Scottish Executive, CZG/1/109 & CZG/4/742), NHS Grampian Endowments (08/02), the European Community's Seventh Framework Programme (FP7/2007-2013) under grant agreement no 212885 and the Medical Research Council, UK (MR/L010011/1). The authors declare they have no competing interests, be it financial, personal or professional.

## Introduction

It is known that anogenital distance (AGD) reflects *in utero* masculinization (Dean and Sharpe, 2013) and in newborn humans AGD is very clearly sexually dimorphic. AGD is being used increasingly as a bio-indicator of fetal androgen exposure in humans and, in particular, to estimate the consequences of adverse *in utero* exposure (e.g. Swan *et al.*, 2005; Kristensen *et al.*, 2011; Mendiola *et al.*, 2011; Castano-Vinyals *et al.*, 2012; Eisenberg *et al.*, 2012; Hsieh *et al.*, 2012; Barrett *et al.*, 2013; Jain and Singal, 2013; Papadopoulou *et al.*, 2013b; Vafeiadi *et al.*, 2013; Mira-Escolano *et al.*, 2014a, b; Thankamony *et al.*, 2014; Adibi *et al.*, 2015; Bornehag *et al.*, 2015; Swan *et al.*, 2015). There is, for example, increasing evidence for a strong link between AGD and reproductive health in men (Eisenberg *et al.*, 2011) and women (Mendiola *et al.*, 2012). There has also been considerable interest in AGD with respect to its gestational correlates, including aspects of fetal/neonatal growth and maternal characteristics (Salazar-Martinez *et al.*, 2004; Sathyanarayana *et al.*, 2010; Papadopoulou *et al.*, 2013a; Barrett *et al.*, 2014). An understanding of normal fetal AGD development in the human is clearly critical, therefore, in order to assess fully the importance and biomedical utility of this parameter (Salazar-Martinez *et al.*, 2004).

We previously published a study that measured AGD in a population of 83 electively terminated, normally progressing human fetuses (11–21 weeks of gestation) (Fowler *et al.*, 2011b). That study showed that AGD is already clearly sexually dimorphic at 11–13 weeks of gestation and that maternal smoking is associated with significantly increased male fetal AGD at 14–16 weeks of gestation. The effect of maternal smoking was surprising given the clear links between maternal cigarette smoking, altered reproductive development and subfertility in adulthood (Jensen *et al.*, 2007; Ramlau-Hansen *et al.*, 2007; Werler, 2007; Fowler *et al.*, 2008). The data reported in (Fowler *et al.*, 2011b) must be considered preliminary, however, in terms of the sample number and there has also been further work reported which extends our knowledge of AGD in humans during the third trimester and up to 24 months post-natally (Thankamony *et al.*, 2009; Gilboa *et al.*, 2014). For these reasons, we have extended our initial study to increase the number of second trimester human fetuses and have attempted to integrate these data into the published literature in order to generate a more complete understanding of changes in human AGD during the fetal and neonatal period.

## Materials and Methods

### Study population

The collection of fetal material (detailed in O'Shaughnessy *et al.*, 2007) was approved by the NHS Grampian Research Ethics Committees (REC 04/S0802/21). Women seeking elective, medical terminations of pregnancy were recruited with full written, informed, consent by nurses working independently at Aberdeen Pregnancy Counselling Service. There was no change in patient treatment or care associated with recruitment to the study and women were able to withdraw from the study at any point. Fetal normality was determined at ultrasound scan 2–9 days prior to the termination of pregnancy. Women bearing abnormal fetuses were not consented for study. Only fetuses from normally progressing pregnancies, from women over 16 years of age with a good understanding of English and between 11 and 21 weeks of gestation, were collected. Fetuses were transported to the laboratory within 30 min of delivery, weighed, crown-rump length (CRL) recorded and sexed (Fowler *et al.*, 2008). Morphologically abnormal fetuses were not included in the study. One gonad was fixed overnight in neutral-buffered formaldehyde, transferred to 70% ethanol and processed for histology. Haematoxylin and eosin-stained gonadal sections were examined to confirm gonadal sex and gross normality.

### Plasma cotinine and testosterone measurement

Cotinine, a metabolite of nicotine and a marker of smoking, was determined in fetal plasma, obtained by cardiac puncture *ex vivo*, using a semi-quantitative commercial assay (Cozart Plc, Abingdon, Kent, UK). Values between 0 and 12 ng cotinine/ml were considered negative (Fowler *et al.*, 2008). In 28 male foetuses, plasma testosterone concentration levels were determined by gas chromatography coupled to tandem mass spectrometry (GC-MS/MS) using the isotope dilution quantification method (Courant *et al.*, 2007, 2010). For testosterone assay, the fetuses were carefully balanced (*n* = 14 controls and 14 smoke-exposed fetuses) with matched fetal age (15.3 ± 2.0 versus 15.4 ± 1.9 weeks of gestation, *P* = 0.907), maternal age (26 ± 2 versus 24 ± 1 years, *P* = 0.747) and maternal body mass index (BMI) (25.8 ± 1.5 versus 24.7 ± 1.1 kg/m^2^, *P* = 0.766) between the control and smoke-exposed groups, respectively.

### AGD (AGD_app_) measurement

**Figure 1:**
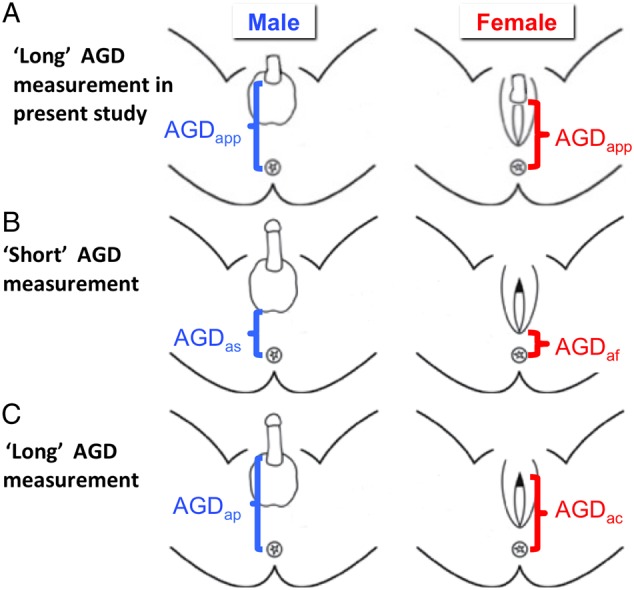
Landmarks for AGD determinations. (**A**) The present study and (**B** and **C**) other published studies in fetuses and neonates. In all cases, the centre of the anus is taken as a common landmark. In males, AGD has been measured as anus to: (A) caudal or posterior insertion of the penis (AGD_app_), (B) scrotum (AGD_as_) or (C) cephalad insertion of the penis (AGD_ap_). In female, AGD has been measured as anus to: (A) caudal or posterior insertion of the clitoris which is relatively large during the second trimester (AGD_app_), (B) base of the posterior fourchette (AGD_af_) and (C) anterior tip of the clitoral hood (AGD_ac_). Redrawn from Salazar-Martinez *et al.* (2004), Thankamony *et al.* (2009), Sathyanarayana *et al.* (2010, 2015), Fowler *et al.* (2011b), Papadopoulou *et al.* (2013a, b), Vafeiadi *et al.* (2013), Barrett *et al.* (2014), Gilboa *et al.* (2014) and Swan *et al.* (2015).

‘Long’ AGD was measured in 126 consecutively collected human fetuses *ex vivo*, from the centre of the anus to caudal or posterior insertion of the penis or clitoris (AGD_app_) using digital callipers (150 mm ISO 9001 electronic calliper, Tesa Technology, Renens, Switzerland) as shown in Fig. 1A and in Fowler *et al.* (2011b). Briefly, for each fetus AGD_app_ was measured as follows: the fetus was laid supine with its legs slightly bent at the knees so that the feet were flat to the dissection surface. By placing the fetus upon laboratory absorbent paper, the fetus remained stable due to slight adhesion of damp skin to the absorbent paper. The fixed calliper point was aligned to the centre of the anus and the moveable point adjusted to line up with posterior insertion of the penis or clitoris and the digital reading recorded. The average of two separate measurements of AGD_app_ was recorded. Over the collection period, three separate researchers recorded AGD_app_ and the spread of AGD_app_ measurements in relation to fetal age was not different between operators. In addition, ANOVA using ‘operator’ as a term to analyse AGD yielded *P* = 0.887. The operators were blinded to maternal smoking status. The reason for using these parameters is that in the younger fetuses measuring ‘short’ AGD anus to scrotum (AGD_as_) or anus to base of the posterior fourchette (AGD_af_) as in (Thankamony *et al.*, 2009; Gilboa *et al.*, 2014) (Fig. 1B) would decrease the accuracy of the measures due to the very small size of the younger fetuses. Subsequently, ‘long’ AGD measurements (Fig. 1C) from anus to cephalad insertion of the penis (AGD_ap_) and anus to the anterior tip of the clitoral hood (AGD_ac_) have been used in neonates (Swan *et al.*, 2015), which could be applied to our second trimester population.

### Real-time quantitative PCR

We have previously found no association between maternal cigarette smoking and testicular anti-apoptotic *BCL2* or pro-apoptotic *BAX* transcript expression in smoke-exposed fetuses (Fowler *et al.*, 2008). In this study, we measured fetal testis transcript expression of: (i) proliferation-associated *PA2G4* (proliferation-associated 2G4, also called *EBP1*), which is also a transcriptional co-repressor of androgen receptor-regulated genes (Lamartine *et al.*, 1997) and (ii) apoptosis-inducing factor mitochondrion associated *AIFM1* (Xie *et al.*, 2005). For quantification of specific mRNA species (see Fowler *et al*., 2009a,b, 2011a) quantitative PCR (qPCR) was used after reverse transcription of isolated RNA (see O'Shaughnessy *et al.*, 2007). The quantity of each measured cDNA from the real-time PCR was expressed relative to the house-keeping gene *TBP* (O'Shaughnessy *et al.*, 2011a,b). The primer sequences are shown in Supplementary data, Table S1.

### Data analysis

JMP 9.0.3 software (Thomas Learning, London, UK) was used. Normality of data distribution was tested with the Shapiro–Wilk test. Non-normally distributed data were examined by Wilcoxon test. Normally distributed data, with log-transformation as required, were analysed by one-way and two-way ANOVA and Tukey–Kramer Honestly Significant Difference post-hoc test. Relationships between morphological measures and weeks of gestation were also explored by linear regression with log transformation as appropriate.

## Results

Measurements of AGD, and supporting data, were collected from 126 elective terminations of normally progressing pregnancies as summarized in Table I. No statistically significant differences in maternal indices were observed between the four groups based upon fetal sex and maternal smoking status.

**Table I:** Characteristics of the pregnancies and fetuses included (mean ± sem).

	Female			Male		
	Control	Smoke-exposed	*P*-value^a^	Control	Smoke-exposed	*P*-value^a^
*N*	32	24	*56*	31	39	*70*
Maternal indices						
Age (years)	25.3 ± 1.2	22.8 ± 1.0	0.135	23.2 ± 1.1	25.4 ± 0.9	0.056
BMI (kg/m^3^)	24.5 ± 0.8	26.0 ± 1.3	0.830	25.0 ± 0.9	25.6 ± 1.0	0.917
Cigarettes/day	0	10.2 ± 0.9		0	10.9 ± 0.9	
Fetal indices						
Weeks of gestation	14.4 ± 0.4	14.6 ± 0.5	0.643	15.1 ± 0.4	15.7 ± 0.3	0.225
Weight (g)	76.2 ± 14.3	76.7 ± 13.0	0.294	87.9 ± 13.2	111.2 ± 12.4	0.123
CRL (mm)	96.6 ± 5.5	95.8 ± 4.5	0.634	102.1 ± 4.8	113.5 ± 4.7	0.092
Paired gonad weight (mg)	18.2 ± 3.1^b^	20.2 ± 3.2	0.440	28.0 ± 3.4^b^	36.5 ± 4.3	0.126
Ponderal index (weight g/[CRL cm^3^])	0.066 ± 0.001	0.070 ± 0.005	0.662	0.069 ± 0.003	0.064 ± 0.002	0.207
AGD measures						
AGD_app_ (mm)^c^	4.87 ± 0.41^b^	4.66 ± 0.33	0.766	9.06 ± 0.77^b^	10.99 ± 0.64	**0.034**
AGD_app_/weight	0.11 ± 0.01^b^	0.09 ± 0.01	0.344	0.14 ± 0.01^b^	0.13 ± 0.09	0.741
AGD_app_/CRL	0.050 ± 0.002^b^	0.048 ± 0.002	0.678	0.086 ± 0.004^b^	0.094 ± 0.003	0.113
AGD_app_/ponderal index	75.6 ± 6.0^b^	68.2 ± 4.5	0.700	135.3 ± 11.2^b^	175.5 ± 12.4	**0.020**

^a^Association with maternal smoking.

^b^Male versus female controls: *P* < 0.05.

^c^AGD_app_ distance (mm) from the centre of the anus to caudal or posterior insertion of the penis or clitoris.

### Sex dimorphism in AGD

**Figure 2:**
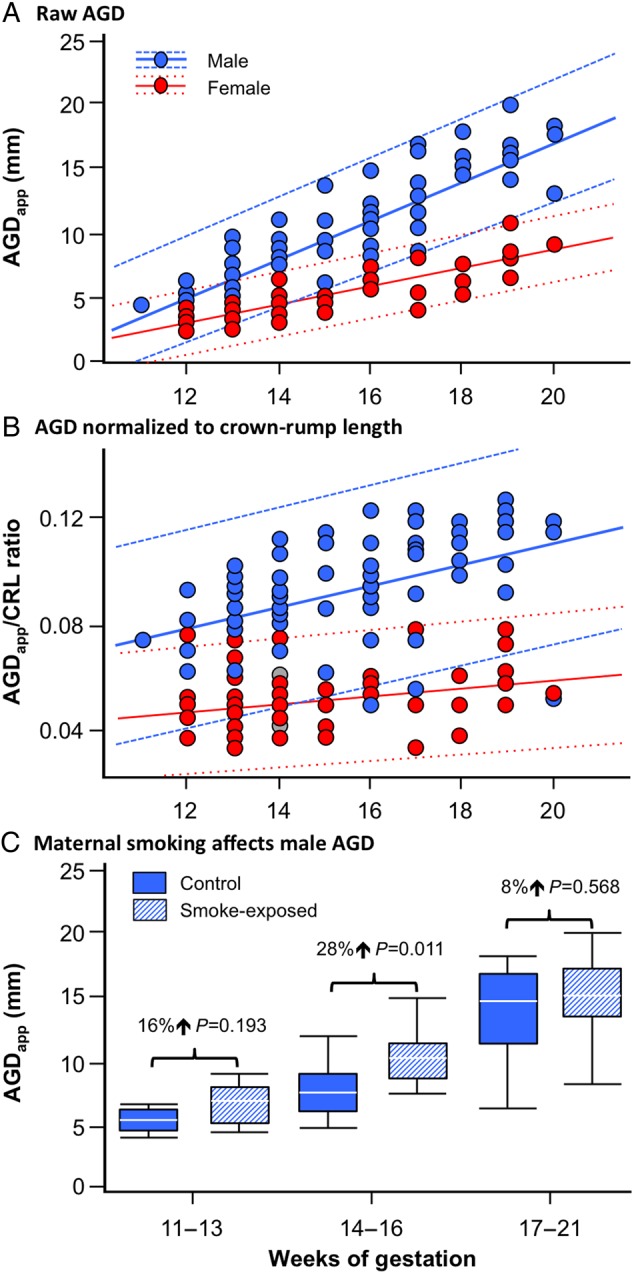
Divergent increase in male and female AGD during the second trimester whether or not growth is included and effects of maternal smoking on male AGD. (**A**) Raw AGD_app_ data (directly measured) and (**B**) AGD_app_ normalized against fetal CRL in second trimester female (*n* = 56) and male (*n* = 70) fetuses, respectively. The solid lines show linear fits and the dotted lines denote the 90% confidence intervals for the linear regressions (A) ♀ AGD_app_ = −5.825 + 0.729*weeks, *P* < 0.001, ♂ AGD_app_ = −13.612 + 1.539*weeks, *P* < 0.001 and (B) ♀ AGD_app_/CRL = 0.0276 + 0.00147*weeks, *P* = 0.045, ♂ AGD_app_/CRL = 0.0279 + 0.00406*weeks, *P* < 0.001. In (**C**), maternal smoking is associated with increased AGD (AGD_app_) in 14–16 week old male fetuses. Data are shown as box and whisker plots in which the horizontal line in the boxes show the median values, with the limits of the boxes showing the 25 and 75% quantiles and the whiskers showing the 10 and 90% quantiles. AGD_app_ distance (mm) from the centre of the anus to caudal or posterior insertion of the penis or clitoris.

Overall, the male fetuses tended to be older than the females but this was not significant for the controls (Table I). This was also reflected by a lack of significant difference between any non-AGD measure other than paired gonad weights, where males had heavier gonads (*P* < 0.01). All measures of AGD (AGD_app_) were significantly (*P* < 0.05–0.001) shorter in females than males. Overall, the rate of increase in AGD_app_, either as raw data (Fig. 2A) or normalized against CRL (Fig. 2B) was slightly higher in males. If the period of study is divided into three developmental windows (Table II), both AGD_app_ and AGD_app_ normalized to ponderal index (an indication of the leanness of the fetus, calculated as: body weight g/[CRL cm^3^]) were significantly shorter in female fetuses at all three periods, with female/male ratios of 61% (*P* < 0.001), 63% (*P* < 0.01) and 70% (*P* < 0.01) at 11–13, 14–16 and 17–21 weeks, respectively.

**Table II:** Comparison of fetal growth between 11–13, 14–16 and 17–21 weeks of gestation (mean ± sem).

Weeks of gestation window	Fetal characteristic	Female		Male	
		Control	Smoke-exposed	Control	Smoke-exposed
11–13	Weeks of gestation (*n*)	12.5 ± 0.2 (16)	12.7 ± 0.2 (10)	12.6 ± 0.4 (9)	12.8 ± 0.2 (6)
	Weight (g)	27.0 ± 2.4	32.3 ± 4.5	28.6 ± 6.2	34.0 ± 6.1
	CRL (mm)	74.6 ± 2.3	78.5 ± 3.4	74.3 ± 3.2	77.8 ± 6.5
	Paired gonad weight (mg)	9.6 ± 1.2^a^	10.4 ± 1.6	17.0 ± 1.6^a^	15.0 ± 1.5
	AGD_app_ (mm)	3.55 ± 0.24^a^	3.47 ± 0.24	5.70 ± 0.56^a^	6.61 ± 0.70
	AGD_app_/ponderal index	56.2 ± 5.5^a^	54.5 ± 3.7	89.1 ± 12.6^a^	98.6 ± 15.2
14–16	Weeks of gestation (*n*)	15.0 ± 0.5 (9)	14.6 ± 0.2 (9)	14.7 ± 0.3 (12)	15.0 ± 0.2 (20)
	Weight (g)	68.0 ± 15.6	68.3 ± 14.4	60.7 ± 6.0	74.4 ± 6.8
	CRL (mm)	99.1 ± 7.2	95.4 ± 2.7	96.8 ± 3.9	103.7 ± 3.2
	Paired gonad weight (mg)	17.4 ± 4.0	17.8 ± 3.6	21.2 ± 2.1	27.5 ± 2.4
	AGD_app_ (mm)	4.65 ± 0.39^a^	4.62 ± 0.33	**7.58 ± 0.63**^a^	**9.73 ± 0.55**
	AGD_app_/ponderal index	76.2 ± 7.2^a^	67.0 ± 6.4	**117.2 ± 11.9**^a^	**153.9 ± 9.7**
17–21	Weeks of gestation (*n*)	18.0 ± 0.5 (7)	18.4 ± 0.4 (5)	17.9 ± 0.4 (10)	18.2 ± 0.3 (13)
	Weight (g)	199.2 ± 29.9	171.9 ± 17.0	174.1 ± 21.2	203.4 ± 15.4
	CRL (mm)	140.3 ± 7.3	131.2 ± 4.8	**133.4 ± 4.5**	**147.8 ± 3.7**
	Paired gonad weight (mg)	42.2 ± 8.9	43.7 ± 5.1	45.2 ± 6.5	62.3 ± 9.1
	AGD_app_ (mm)#	8.16 ± 0.94^a^	7.08 ± 0.51	13.85 ± 1.17^a^	14.97 ± 0.89
	AGD_app_/ponderal index	116.3 ± 11.7^a^	94.7 ± 7.8	198.6 ± 16.2^a^	249.9 ± 21.8

AGD_app_, anogenital distance (mm) measured from the centre of the anus to caudal or posterior insertion of the penis or clitoris.

^a^Male versus female controls: *P* < 0.05; values in bold within each sex: *P* < 0.05.

### Effect of maternal cigarette smoking on fetal AGD

Maternal cigarette smoking had no significant association with female fetal AGD_app_ using the unprocessed data (Table I) or following separation in developmental windows (Table II). In contrast, both unprocessed AGD_app_ and AGD_app_ normalized against ponderal index were significantly (*P* < 0.05) increased in smoke-exposed males (Table I). When data were separated into three developmental windows (Table II), it was clear that at 14–16 weeks of gestation, maternal smoking was associated with significantly (*P* < 0.05) increased AGD_app_ and AGD_app_, normalized against ponderal index. Figure 2C shows smoke-exposed versus control divergence in AGD_app_ with the relative difference between control and smoke-exposed fetuses largest at 14–16 weeks of gestation. No significant difference in variance was observed between groups (Levene's Test). Further analysis by two-way ANOVA (sex, smoking, gestational age) confirmed interactions between sex and weeks of gestation (*P* < 0.001) and between sex and smoking (*P* < 0.01).

### Testosterone and proliferation/apoptosis in relation to fetal AGD

**Figure 3:**
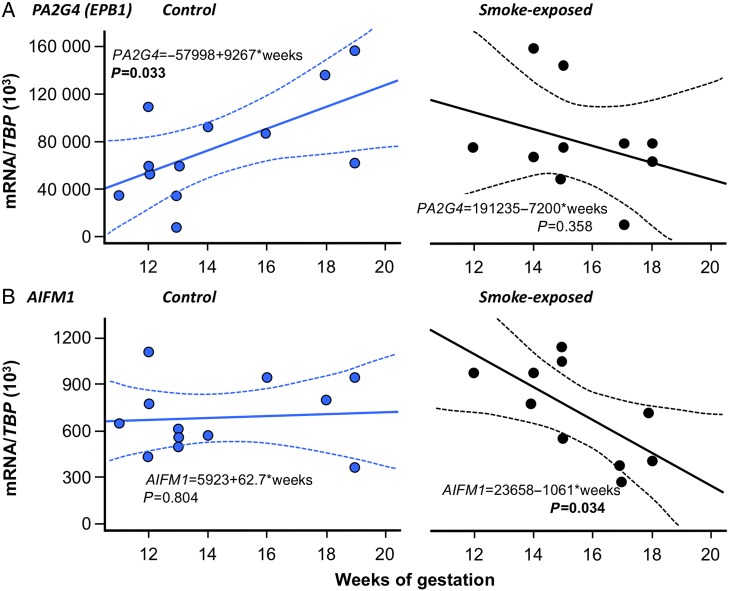
Maternal cigarette smoking is associated with divergent changes in testis expression of transcripts for *PA2G4* and *AIFM1*. In the case of (**A**) *PA2G4*, a significant positive association between expression and fetal age is reversed by smoke-exposure while for (**B**) stable *AIFM1*, expression is altered to a significant negative association between expression and fetal age. The solid lines show linear fits and the dotted lines denote the 90% confidence intervals for the linear regressions.

We have previously reported that maternal smoking is not associated with significantly altered male fetal human plasma testosterone (by DELFIA assay: (O'Shaughnessy *et al.*, 2007; Fowler *et al.*, 2008)). In the current study, male fetal plasma testosterone (measured using GC-MS/MS) was similar in both control and smoke-exposed fetuses (1.77 ± 0.28 ng/ml in controls versus 1.80 ± 0.34 ng/ml in smoke-exposed, *P* = 0.646, see Table III). In 21 of these male fetuses, AGD data were also available but we found no statistically significant correlation between concurrent circulating testosterone and either raw AGD or AGD normalized by BMI, CRL or ponderal index. It should be noted that the spread of fetuses across the whole age range reduces the magnitude of the difference in AGD associated with maternal smoking. Taken together with our previously published data, this shows that smoke-exposure is not associated with any change in the profile of decreasing testosterone levels across the second trimester. There was no significant effect of smoke exposure on transcript levels of either *PA2G4* (74,832 ± 12,589 versus 79,631 ± 13,674 (/*TBP* × 10^3^), *P* = 0.575) or *AIFM1* (6821 ± 655 versus 7207 ± 981(/*TBP* × 10^3^), *P* = 0.767). However, maternal smoking was associated with significant changes in the developmental trajectory of these transcripts across the second trimester (Fig. 3). Specifically, the statistically significant trend for increasing testis expression of *PA2G4* is lost in smoke-exposed fetuses (Fig. 3A) while the trend for stable testis *AIFM* expression across the second trimester becomes a statistically significant trend for reduced expression if the mother smokes (Fig. 3B).

**Table III:** Maternal smoking is not associated with differences in the relationship between plasma testosterone and AGD_app_ in male fetuses (mean ± sem).

Fetal characteristic	Control	Smoke-exposed
Plasma testosterone (ng/ml)	1.77 ± 0.28	1.80 ± 0.34
AGD_app_ (mm)^a^	8.64 ± 1.05	9.75 ± 0.97

^a^AGD_app_ distance (mm) from the centre of the anus to caudal or posterior insertion of the penis or clitoris.

### Integrating AGD studies from fetal to early post-natal life: developmental trajectories of AGD

**Figure 4:**
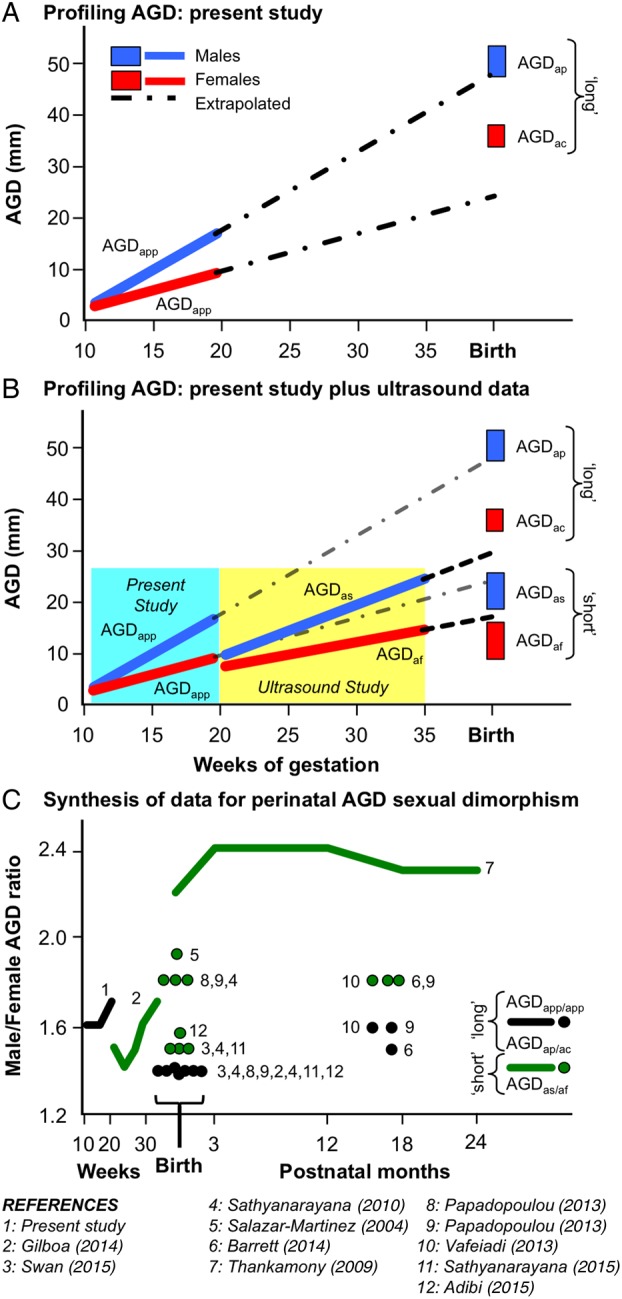
Integration of AGD data from selected published studies. AGD_app_ was directly measured *ex vivo* in fetuses at 11–21 weeks of gestation. Ultrasound was used to determine AGD_as_ and AGD_af_ *in vivo* in fetuses between 20 and 35 weeks of gestation. Direct *in vivo* measurements of neonates and infants were used to determine AGD_ap_ and/or AGD_as_ in males and AGD_af_ and/or AGD_ac_ in females. In (**A**), the changes in male and female AGD_app_ are shown from 10 weeks of gestation up to birth. The lines denote trajectories while the boxes show the range of mean AGDs from different studies at birth (AGD_ap_ and AGD_ac_). In (**B**), the data shown in (A) are overlaid with data from *in vivo* measurement of AGD by ultrasound (AGD_as_ and AGD_af_). In (**C**), the female/male AGD ratios are shown. The lines join either repeated measures of the same individuals or measurements across individuals of different ages while circles show single or two time-points only. The 11 studies shown in the key for (C) also contributed the ‘long’ and ‘short’ AGD data shown in (A and B).

In Fig. 4, data on AGD_app_ from this study are integrated with data from other studies reporting AGD measurements between 20 weeks of gestation and 24 months post-natal. When both AGD_as_ and AGD_ap_ were measured in males and AGD_af_ and AGD_ac_ were measured in females, the within-sex ratios between the two measures (i.e. using different landmarks, see Fig. 1) were reasonably consistent (1.9–2.3 for males and 1.9–2.5 for females). Therefore, in terms of the trajectories of increasing AGD with gestational age and at birth (Fig. 4A) the AGD_as_ and AGD_af_ and AGD_ap_ and AGD_ac_ values ended at similar points when these ratios were used as conversion factors. It is clear that from 10–13 weeks of gestation up to birth, AGD increases approximately linearly, with male AGD increasing along a steeper slope. However, some interesting detail emerges with the *in utero* ultrasound data overshooting cognate, landmark-method, neonatal AGD (AGD_as_, AGD_af_) as shown in Fig. 4B. In contrast, when linearly extrapolated, our *ex vivo* second trimester data showed close similarity to neonatal AGD_ap_ in males, whereas in females the extrapolation markedly undershot neonatal AGD_ac_ values.

### Integrating AGD studies from fetal to early post-natal life: sexual dimorphism in AGD

We further interrogated our data and the literature by calculating the ratio of male:female AGD (male AGD/female AGD), i.e. the larger the value, the greater the sexual dimorphism in AGD (Fig. 4C). Apart from Thankamony *et al.* (2009), all the male/female AGD ratios fell between 1.4 and 1.9 and the AGD_as_/AGD_af_ sex dimorphism ratios were higher than the AGD_ap_/AGD_ac_ ratios. What is also apparent, however, from the combined data are that the study/methodology/landmark selection is a greater determinant of the male/female AGD ratio than fetal or neonatal age (Fig 4C).

## Discussion

Use of AGD as an index of masculinization and, hence, exposure to androgen in fetal life may be a particularly powerful way of linking fetal reproductive programming to adult disease. To fulfil this potential, however, it is essential that normal population data are generated as argued recently (Dean and Sharpe, 2013). In this study, we expand the population size of our previous study (Fowler *et al.*, 2011b) (from 83 to 126 fetuses) and also incorporate the data from 11 other published studies on fetal, neonatal and early infancy AGD in both male and female offspring. The combined data now provide a framework showing changes in AGD across the entire sexually dimorphic period of human gestation. Results from the expanded study confirm that: (i) the male fetus already has a significantly greater AGD than the female at 11–13 weeks of gestation, (ii) that there is a linear increase in AGD in both sexes throughout gestation and (iii) that maternal smoking is associated with a transient increase in male AGD during the second trimester. The 53 additional fetuses combined with our previous study data (Fowler *et al.*, 2011b) have served to increase both the magnitude and statistical significance of the effect of maternal smoking on AGD in male fetuses at 14–16 weeks, adding confidence that this is a consistent observation. Reviewing these results in the context of other published data also shows, however, that considerable variation exists across studies in certain measured parameters which may limit use of AGD measurements unless more standardized procedures can be developed.

One surprising finding identified in Fowler *et al.* (2011b) was that maternal smoking was associated with significantly increased AGD in male fetuses, but not in females, during the middle part of the second trimester. Our expanded dataset (Table II) has strengthened the significance of this observation with a 28% increase in male smoke-exposed AGD at 14–16 weeks. The findings with respect to smoke-exposure in other gestation age groups have remained very similar between the original study and the updated data in Fig. 1B. This finding was surprising since it suggests a dysregulation of masculinization in the period following peak testosterone in the male fetus (O'Shaughnessy *et al.*, 2007), with the dysregulation corrected by the end of the second trimester. This suggests that androgen-dependent growth of the anogenital area is altered in these smoke-exposed fetuses. We have previously found adverse and sex-specific outcomes associated with maternal cigarette smoking during pregnancy on a number of developmentally important organ systems (e.g. testis, ovary, liver (Fowler *et al.*, 2008, 2009a,b), (Fowler *et al.*, 2014) (O'Shaughnessy *et al.*, 2011a,b, 2013; Drake *et al.*, 2015; Filis *et al.*, 2015)) and changes in AGD may, therefore, be part of a general trend. A theme common to these tissues is that there is evidence for dysregulation (sex-dependent in the case of the liver) of the progression of organ development if the mother smokes while pregnant and at least one pathway through which smoking may affect development is via the aryl hydrocarbon receptor (AHR), at least in the ovary (Anderson *et al.*, 2014; Fowler *et al.*, 2014). The subsequent reversal of the effects of smoking on AGD in the male may be because of tissue plasticity which has been shown (Gyekis *et al.*, 2010; Mitchell *et al.*, 2015), at least in rodents, to allow recovery of AGD from adverse effects.

The increase in AGD in smoke-exposed males we report here is occurring just after the peak time of androgen action on the AGD in the male fetus. Maternal smoking does not appear to alter fetal testosterone levels, however, during the second trimester (this study and Fowler *et al.*, 2008) and so smoke-induced changes in AGD appear unlikely to be due to direct changes in androgen levels. It is possible, however, that circulating testosterone levels may not be the most relevant indicator of androgen exposure to the external genitalia since it has been shown that the alternative, ‘backdoor’ pathway of androgen synthesis may be important in the human (Fluck *et al.*, 2011). More comprehensive analysis of circulating androgens and maternal smoking effects in the human male fetus would therefore be required in order to more comprehensively characterize the impact of smoking on steroidogenesis. Alternatively, maternal smoking may act on the AGD of male fetuses directly through interaction with the effects of androgen or other endocrine systems. This may, for example, be through effects on expression or activity of the androgen receptor in the region of the external genitalia. Maternal smoking is associated with sex-specific alterations in hCG levels (Fowler *et al.*, 2009a,b, 2014; Varvarigou *et al.*, 2009) and hCG has been linked to adverse effects induced by some environmental chemical contaminants, such as phthalates (Adibi *et al.*, 2015). Changes in the gonadotrophin drive may, therefore, have effects on AGD that do not depend on androgen as an intermediary. Cigarette smoke also contains activating ligands for the AHR (e.g. polycyclic aromatic hydrocarbons) and these have been shown to be amongst the active compounds which affect female reproductive development in rodents (Matikainen *et al.*, 2001). Similarly, changes in fetal AHR signalling have been shown to be associated with adverse effects in the human fetal ovary (Anderson *et al.*, 2014; Fowler *et al.*, 2014). While effects of AHR stimulation remain to be established in androgen-responsive tissues such as the external genitalia it is possible that the AHR system is involved in the association between maternal cigarette smoking and altered AGD.

We have, for the first time, characterized *PA2G4* and *AIFM1* expression in the human fetal testis to expand our understanding of the association between maternal smoking and the balance between proliferation and apoptosis in fetal tissues. For both transcripts maternal smoking acted to alter the normal developmental trajectory, which might be expected to change the dynamics of tissue growth. Reduced expression of *AIFM1* caused by maternal smoking would, for example, be indicative of a reduction in apoptosis with potential adverse consequences, including reduced protection from chemical-stress induced apoptosis (Urbano *et al.*, 2005) by AHR activating ligands (Coutts *et al.*, 2007). Reduced *AIFM1* is also associated with disturbed oxidative phosphorylation (Vahsen *et al.*, 2004), suggesting another pathway to tissue damage in smoke-exposed fetuses. In the case of PA2G4, changes in expression may lead to altered growth regulation and to a potential reduction in control of androgen signalling (Zhang and Hamburger, 2005; Zhou *et al.*, 2010). The pattern of transcript dysregulation described here is similar to that shown between maternal smoking and ovarian transcript signalling with coincident alterations in ovarian morphology (Fowler *et al.*, 2014). Since maternal smoking is associated with reduced fetal and placental growth, it may also be pertinent that *PA2G4* and *AIFM1* are widespread in tissues and *PA2G4*-null and *AIFM1*-null mice show significant growth reduction (although embryo lethal by GD9 in the case of *AIFM1*) (Brown *et al.*, 2006; Zhang *et al.*, 2008).

Currently, published studies are lacking on the association between maternal cigarette smoking, or other adverse maternal circumstances (alcohol consumption, diet, obesity and deprivation), and the AGD of offspring either post-natally or in adulthood. Therefore, further robust AGD measures and their integration with reproductive and health indices across life are required. In previous studies (Thankamony *et al.*, 2009; Gilboa *et al.*, 2014), non-invasive approaches were used to quantify AGD in the third trimester of pregnancy and in the neonate/infant and this is probably the best way to develop this normative data. We suggest that studies should be extended to include the effects of the maternal environment on the range of the normative data across pre-, peri- and post-natal development.

Integration of data already available (Fig. 4A) shows that AGD increases in a linear fashion throughout the second and third trimesters of gestation in both sexes. However, there are sex-specific differences in this pattern during fetal life. The growth in male fetal AGD from 11 to 21 weeks of gestation that we present here is linear, intersecting with the range of landmark cognate measures of AGD in neonates from several studies. In contrast, the equivalent AGD measure in our female fetuses undershoots the expected neonatal AGD_ac_ from several studies by some 10 mm. Given that we do not see a marked difference in overall fetal weight and length in our control fetuses (Table II), the reason for this is not clear. Some discrepancy between *in utero* ultrasound (Gilboa *et al.*, 2014) and *in vivo* post-natal AGD measurement would also be expected because of the very different methodologies used to make the measurements. However, when extrapolated, the ultrasound AGD growth trajectory only deviates slightly from expected neonatal AGD_as_ and AGD_af_ measures from a number of studies. Taken together, this suggests that there is a relative increase in female fetal AGD growth rates at some point in the second half of pregnancy although there is insufficient mechanistic data to do more than speculate about potential mechanisms.

To gain a better understanding of the growth trends in AGD, we plotted the male/female AGD ratio from 11 studies including the present study (Fig. 4C). When these data are presented together, there is clear variability in the male/female AGD ratio between studies and between landmarks used. The most consistent difference is that the AGD_as_/AGD_af_ ratio is higher (i.e. more masculinized) for a given age than the AGD_ap_/AGD_ac_. This higher AGD_as_/AGD_af_ ratio may be an indication that the whole AGD is not equally sexually dimorphic with a greater effect of androgens seen in the region of the perineum. Inter-study variability is most clearly seen in the reported male/female AGD_as_/AGD_af_ ratio at birth. For example, there is a 60% difference in male/female AGD ratio (Sathyanarayana *et al.*, 2010, 2015; Swan *et al.*, 2015) and (Thankamony *et al.*, 2009; Adibi *et al.*, 2015). The reason for this is not clear—a primary cause could simply be ethnic and technical variation. This is supported by the fact that in their more recent publication on boys only (Thankamony *et al.*, 2014), the mean male AGDs were 3 mm shorter on average at 12 months post-natal age than in Thankamony *et al.* (2009). Therefore, small sex-specific variation in AGDs between studies could manifest as quite large differences in male/female AGD ratios. It is also noticeable that in Thankamony *et al.* (2009) males were slightly heavier and longer at birth (and subsequently) than females, whereas in other studies (e.g. Salazar-Martinez *et al.*, 2004) there was no sexual dimorphism in birth size. While this makes no difference in terms of within study analyses, an inevitable conclusion is that in order to allow more robust inter-study comparisons, some effort must be made to find methodologically and statistically robust methods to normalize AGD against fetal/neonatal/infant size. It should also be stated that all the studies of AGD with a neonatal measurement and at least one other time point in infancy are internally consistent, with very little evidence for any further change in the male/female AGD ratio, even though androgen action is clear during the post-natal mini-puberty (e.g. Pasterski *et al.*, 2015).

In conclusion, this study shows that second trimester human fetal AGD growth is sexually dimorphic and linear and confirms that in male fetuses maternal cigarette smoking is associated with an unexpected and temporary effect of increasing AGD. Overall, given the well-established link between AGD and defects in reproductive development (e.g. hypospadias (Thankamony *et al.*, 2014)), the techniques and study populations are now available for much more informative studies to be carried out across gestation and in the perinatal/post-natal period. This will enable a much-improved understanding of the links between maternal environment/lifestyle and fetal and neonatal reproductive and functional development. The introduction of ultrasound scan technologies to these studies will enable us to examine more deeply into the role of the intrauterine environment and to follow-up studies in limited numbers of fetuses with properly powered non-invasive studies.

## Supplementary data

Supplementary data are available at http://humrep.oxfordjournals.org/.

## Authors' roles

P.A.F. conception, design, analysis, interpretation, drafting, revision, approval. P.F. acquisition, analysis, revision, approval. A.J.D. drafting, revision, approval. S.B. conception, design, drafting, revision, approval. J.-P.A. analysis, drafting interpretation, approval. B.B. interpretation, revision, approval. M.-L.M. analysis, interpretation, approval. U.S. analysis, interpretation, approval. P.J.O.S. conception, design, analysis, interpretation, drafting, revision, approval.

## Funding

Support for the study was provided by the Chief Scientist Office (Scottish Executive, CZG/1/109 & CZG/4/742), NHS Grampian Endowments (08/02), the European Community's Seventh Framework Programme (FP7/2007-2013) under grant agreement no 212885 and the Medical Research Council, UK (MR/L010011/1). Funding to pay the Open Access publication charges for this article was provided by the Medical Research Council.

## Conflict of interest

None declared.

## Supplementary Material

Supplementary Data
