# Supplementary material for: Human anogenital distance: an update on fetal smoke-exposure and integration of the perinatal literature on sex differences
Source: Hum Reprod. 2016 Jan 4;31(2):463–72. doi: 10.1093/humrep/dev323 (PMC4716811; doi:10.1093/humrep/dev323)
Supplement: Supplementary Data [file supp_31_2_463__index.html]

Human anogenital distance: an update on fetal smoke-exposure and integration of the perinatal literature on sex differences — Human anogenital distance: an update on fetal smoke-exposure and integration of the perinatal literature on sex differences — Supplementary Data 

# Human anogenital distance: an update on fetal smoke-exposure and integration of the perinatal literature on sex differences

## Supplementary Data

Supplementary Data

- Supplementary Table 1 - pdf file
