# Supplementary material for: Human anogenital distance: an update on fetal smoke-exposure and integration of the perinatal literature on sex differences
Source: Hum Reprod. 2016 Jan 4;31(2):463–72. doi: 10.1093/humrep/dev323 (PMC4716811; doi:10.1093/humrep/dev323)
Supplement: Supplementary Data [file supp_dev323_dev323supp_table1.pdf]

Human Reproduction
doi:10.1093/humrep/dev323

human reproduction

SUPPLEMENTARY DATA

Supplementary Table SI Primers used for qPCR.

Gene symbol	Gene name	GenBank	Forward primer	Reverse primer
PA2G4	Proliferation-associated 2G4, 38kDa	NM_006191.2	tggtcccttcgagcctgacct	tccccactggtggcattctctg
AIFM1	Apoptosis-inducing factor, mitochondrion-associated, 1	NM_004208.3	cccgaatagcggtcgccgaa	aggaacatgccatcgctggaaca
TBP	TATA box binding protein	NM_003194	aggaaaaaattgaatagtgagacgagttcca	tggactaaagatagggattccgggagt
